# Adenovirus E1B-55K interferes with cellular IκB kinase complex subunit proteins

**DOI:** 10.3389/fimmu.2025.1532742

**Published:** 2025-03-04

**Authors:** Wing-Hang Ip, Luca D. Bertzbach, Sabrina Schreiner, Thomas Dobner

**Affiliations:** ^1^ Department of Viral Transformation, Leibniz Institute of Virology, Hamburg, Germany; ^2^ Institute of Virology, Medical Center, University of Freiburg, Freiburg, Germany; ^3^ Institute of Virology, Hannover Medical School, Hannover, Germany; ^4^ Cluster of Excellence RESIST (Resolving Infection Susceptibility; EXC 2155), Hannover Medical School, Hannover, Germany

**Keywords:** E1B-55K, HAdV-C5, human adenovirus, IKKα, IKKβ, NEMO, NF-κB pathway, TNFα

## Abstract

Human adenovirus (HAdV) infections can cause high mortality rates in immunocompromised patients due to the activation of unhampered cytokine storms that are mainly induced by activation of pro-inflammatory cytokines. NF-κB is a transcription factor that is involved in numerous biological processes such as regulation of cell death and proliferation, as well as the activation of innate immune responses including the expression of pro-inflammatory cytokines, chemokines, and other immune response genes. The IKK complex plays a crucial role in the NF-κB pathway by phosphorylating and activating IκB proteins, which leads to the degradation of IκB and the subsequent release and nuclear translocation of NF-κB dimers to initiate gene transcription. The host NF-κB pathway, particularly the formation of the IKK complex, is a common target for viruses to regulate host immune responses or to utilize or inhibit its function for efficient viral replication. So far, investigations of the immune response to adenovirus infection mainly focused on transduction of adenoviral vectors or high-titer infections. Therefore, the molecular mechanism of HAdV- and HAdV gene product-mediated modulation of the NF-κB response in lytic infection is not well understood. Here, we show that HAdV-C5 infection counteracts cellular IκB kinase complex formation. Intriguingly, the IKK complex protein IKKα is targeted to the nucleus and localizes juxtaposed to viral replication centers. Furthermore, IKKα interacts with the early viral E1B-55K protein and facilitates viral replication. Together, our data provide evidence for a novel HAdV-C5 mechanism to escape host immune responses by utilizing NF-κB pathway-independent nuclear functions of IKKα to support efficient viral progeny production.

## 1 Introduction

The innate immune response is directly activated upon pathogen recognition and provides the first line of defense by generating an immediate, non-specific response against pathogen components (1). Some viruses, however, exploit this mechanism to their advantage. For example, some have binding sites in their promoters that are specifically recognized and activated by immune response-regulating transcription factors like NF-κB. This allows these viruses to hijack the host’s immune response, promoting their own replication and gene expression (2). The NF-κB signaling pathway plays a key role in regulating cellular survival and death, responding to various stimuli through the phosphorylation of the IκB kinase (IKK) complex. Viruses, particularly DNA viruses, modulate this pathway to enhance their replication and establish infections. By interacting with components of the NF-κB cascade, viruses either activate or inhibit the pathway to create an environment conducive to productive infection (2, 3). Understanding these viral interactions offers insights into infection-related pathogenesis and potential therapeutic targets (4).

The NF-κB transcription factor represents a group of evolutionarily conserved and structurally related proteins belonging to the Rel protein family (5). So far five NF-κB proteins, NF-κB1 (a.k.a. p50/p105) and NF-κB2 (a.k.a. p52/p100), RelA (a.k.a. p65), RelB and c-Rel, have been described. The NF-κB proteins are only transcriptionally active as a dimer, but some combinations are thought to act as inactive or repressive complexes (6–9). The classical heterodimeric NF-κB transcription factor, which is usually referred to, is the abundant expressed dimer of p50 and RelA (p65), and it has been the most intensively studied dimer of the NF-κB pathway (5).

A wide variety of cellular signals can activate the NF-κB pathway upon ligand binding. This activation involves the IKK complex proteins, which play a key role in the signaling cascade. Two main signaling pathways activate NF-κB; the canonical and the non-canonical pathway (10, 11). It has been shown that both pathways have different regulatory functions: the canonical pathway is mostly involved in innate immunity, whereas the non-canonical pathway regulates the development of lymphoid organs and the adaptive immunity (10, 12). However, activation of both signaling pathways of NF-κB converge at the activation of IKK. Therefore, it is the most important regulatory component of the pathway and activates NF-κB through two distinct mechanisms (5). The IKK kinase complex is a 700-900 kDa complex containing the catalytic subunits IKKα [a.k.a. conserved helix-loop-helix ubiquitous kinase (CHUK)] and IKKβ, and the regulatory subunit IKKγ (a.k.a. NF-κB essential modifier or NEMO, IKKAP1, and Fip-3) (5, 10). The canonical NF-κB pathway catalyzes the phosphorylation of IκBs mainly in a IKKβ- and NEMO-dependent manner; while the non-canonical NF-κB pathway is strictly activated through IKKα and is independent of IKKβ and NEMO. Key receptors that can activate NF-κB include cytokine receptors such as IL-1R and TNF-α receptors, toll-like receptors (TLRs), antigen receptors, and various other signaling receptors. TNFα, for example, activates NF-κB by stimulating the IKK complex, which leads to the phosphorylation and degradation of IκB, releasing NF-κB to translocate into the nucleus and activate target genes.

Human adenoviruses (HAdVs) have developed diverse strategies over the course of evolution to modulate both innate and adaptive immune responses at the molecular level, mainly through proteins encoded in the E3 region (13). This modulation is particularly evident in how HAdV regulates TNFα signaling. Studies on adenoviral vectors that do not express the viral proteins inhibiting the function of TNFα have shown that these vectors are inactivated by TNFα. As a result, the expression of transgenes encoded within the adenoviral vectors is shortened (14). During infection, the early viral protein E1A sensitizes cells to TNFα-induced apoptosis, which is subsequently counteracted by the E1B-19K protein (15, 16). Numerous studies have shown that adenoviral vectors can induce cell line-dependent cytokine expression via NF-κB activation (17). However, the molecular mechanism by which NF-κB is modulated during lytic infection remains largely unknown.

Here, we aimed to investigate the regulation of IKK complex formation during the late phase of HAdV-C5 infection. We demonstrated that this regulation is inhibited, which leads to the repression of TNFα-induced NF-κB activation. E1B-55K plays a role in immune pathway deregulation (18). It is well known that E1B-55K is a multifunctional protein that, for example, can relocalize proteins or, in conjunction with the viral protein E4orf6, target proteins for proteasomal degradation (19, 20). Therefore, we wanted to investigate how E1B-55K, as an adenoviral regulatory protein, interferes with immune pathways, similar to how other viruses use their regulatory proteins. In this work, we demonstrated that HAdV infection induces relocalization of IKKα to the nucleus and showed data that indicate that E1B-55K suppresses TNFα-induced immune responses mediated by the NF-κB pathway during late infection stages. Additionally, NEMO is also relocalized to viral replication centers independently of E1B-55K. Furthermore, IKKα was found to support the production of progeny viruses. In summary, our findings suggest that HAdV-C5 specifically interferes with IKK complex proteins while simultaneously exploiting the NF-κB-independent functions of IKKα and NEMO.

## 2 Materials and methods

### 2.1 Cell culture and generation of knockout cell lines

H1299 cells (ATCC CRL-5803) and A549 cells (ATCC CCL-185) were grown in Dulbecco’s modified Eagle’s medium (DMEM; Sigma) supplemented with 10% fetal calf serum (PAN Biotech) and 100U of penicillin/100 µg of streptomycin per ml (PAN Biotech) in a 5% CO_2_ atmosphere at 37°C. IKKα knock-down cell lines were generated by lentiviral transduction using recombinant lentiviral particles expressing shRNA targeted to IKKα (5’-ACA GCG TGC CATTGA TCT ATA-3’; Mission RNA Sigma-Aldrich). All cell lines were regularly tested for mycoplasma contamination.

### 2.2 Plasmids and transient transfections

For transient transfections, subconfluent cells were treated with a mixture of DNA and 25 kDa linear polyethylenimine (PEI; Polysciences), following a recently described protocol (21). Please refer to **Table 1** for information about the plasmids.

**Table 1:** Plasmids.

Name	Vector	Insert
E1A	pGL3	HAdV-C5 E1A promoter reporter gene construct
E1B	pGL3	HAdV-C5 E1B promoter reporter gene construct
E1B-55K	pcDNA3	HAdV-C5 E1B-55K
E1B-55K delP	pcDNA3	HAdV-C5 E1B-55K
E1B-55K NES	pcDNA3	HAdV-C5 E1B-55K
E1B-55K SUMO	pcDNA3	HAdV-C5 E1B-55K
E1B-55K-pM	pcDNA3	HAdV-C5 E1B-55K
E2	pcDNA3	HAdV-C5 E1B-55K
E2E	pGL3	HAdV-C5 E2 early promoter reporter gene construct
E2L	pGL3	HAdV-C5 E2 late promoter reporter gene construct
E3	pGL3	HAdV-C5 E3 promoter reporter gene construct
E4orf6	pcDNA3	HAdV-C5 E4orf6
EE	pcDNA3	HAdV-C5 E1B-55K
Flag-IKKα	pCR-Flag	Flag-tagged IKKα
Flag-IKKβ	pCMV2	Flag-tagged IKKβ
Flag-NEMO	pCMV-Flag	Flag-tagged NEMO
H260A	pcDNA3	HAdV-C5 E1B-55K
HA-NEMO	pcDNA3-HA	HA-tagged NEMO
L4-100K	pCR	HAdV-C5 L4-100K
NF-κB	pGL3	5x NF-κB (binding sites) with ELAM-promoter
R443A	pcDNA3	HAdV-C5 E1B-55K
R443ins	pcDNA3	HAdV-C5 E1B-55K
Renilla	pRL-TK	Renilla under the control of the HSV-TK promoter
RF6	pcDNA3	HAdV-C5 E1B-55K
RTR	pcDNA3	HAdV-C5 E1B-55K

### 2.3 Viruses

H5*pg*4100 was used as the wild-type (wt) virus with a cell culture dispensable deletion (nt 28593-30471) in the E3 region (22). The mutant virus H5*pm*4149 contains stop codons in the E1B-55K open reading frame, preventing its expression (23). All viruses were propagated and titrated as previously described (24). For virus yield experiments, infected cells were harvested 24 and 48 hours post-infection (h p.i.), lysed by three freeze-thaw cycles, and then reinfected into cells for 24 h. Virus growth was assessed by immunofluorescence staining for the adenoviral DNA-binding protein DBP/E2A.

### 2.4 Luciferase assays

H1299 cells were co-transfected with a Firefly luciferase plasmid, controlled by either an NF-κB-responsive promoter (5x NF-κB-ELAM-promoter) or viral promoters from early transcription units (E1A, E1B, E2E, E2L, and E3), along with effector plasmids for 24 h (**Table 1**). To control for transfection efficiency, a Renilla luciferase plasmid was co-transfected and used for normalization. For NF-κB promoter activation following TNFα treatment, H1299 cells were first transfected with the Firefly luciferase plasmid under the control of the NF-κB promoter. 22 h after transfection, the cells were treated with TNFα (PeproTech) for 2 h, then immediately infected at the indicated multiplicity of infection (MOI, defined as ffu/cell) for up to 24 h. For measurements, the cells were lysed and analyzed using the Dual-Luciferase Reporter Assay System (Promega), with sequential measurement of Firefly and Renilla luciferase activity.

### 2.5 Protein analyses and immunoprecipitation

All protein extracts were prepared using radioimmunoprecipitation assay (RIPA) lysis buffer, as previously described and protein concentration was determined (25). For immunoprecipitation, protein A-sepharose beads (Sigma-Aldrich Inc.) were coupled with 1 µg of monoclonal antibody for 1 h at 4°C (3 mg per immunoprecipitation). The antibody-coupled protein A-sepharose was then added to precleared pansorbin-sepharose extracts (50 µl per lysate; Calbiochem) and rotated for 2 h at 4°C. Proteins bound to the antibody-coupled protein A-sepharose were precipitated by centrifugation, washed three times. Immunoprecipitation and input control samples, with the input controls adjusted to the same protein concentration, were boiled in Laemmli buffer at 95°C for 3 minutes and then analyzed by immunoblotting as described (26). Please refer to **Table 2** for all primary antibodies. Secondary antibodies conjugated to horseradish peroxidase (HRP) for protein detection via immunoblotting included anti-rabbit IgG, anti-mouse IgG, and anti-rat IgG (Jackson/Dianova).

**Table 2:** Antibodies.

Antibody^1^	Concentration^1^	Company or reference
Mouse mAb 2A6 (E1B-55K)	1:10 (WB) 1:10 (IF)	(79)
Mouse mAb AC-15 (β-actin)	1:5000 (WB)	Sigma-Aldrich
Mouse mAb B6-8 (DBP)	1:10 (WB) Undiluted (IF)	(80)
Mouse mAb DO-1 (p53)	1:1000 (WB)	Santa Cruz
Mouse mAb M2 (Flag)	1:2000 (WB)	Sigma-Aldrich
Mouse mAb M73 (E1A)	1:10 (WB)	(81)
Mouse mAb RSA3 (E4orf6)	1:10 (WB)	(82)
Polyclonal rabbit pAb (HAdV-C5 E1B-19K)	1:3000 (WB)	(83)
Rabbit pAb (IKKα)	1:3000 (WB) 1:50 (IF)	Santa Cruz
Rabbit pAb (IκBα)	1:1000 (WB)	Santa Cruz
Rabbit pAb (NEMO)	1:1000 (WB) 1:50 (IF)	Santa Cruz
Rabbit pAb pNB 100-142 (Mre11)	1:1000 (WB)	Novus Biologicals
Rabbit polyclonal serum L133 (HAdV capsid)	1:10000 (WB)	(23)
Rat mAb 3F10 (HA)	1:1000 (WB)	Roche
Rat mAb 6B-10 (L4-100K)	1:20 (WB)	(84)
Rat mAb 7C11 (E1B-55K)	1:10 (WB)	(23)

^1^mAB, monoclonal antibody; pAb, polyclonal antibody; WB, western blotting; IF, immunofluorescence.

### 2.6 Expression and purification of recombinant fusion proteins (GST pulldown)

E1B-55K fragments tagged with glutathione-S-transferase (GST) in pGEX vectors (27, 28) were cultured in LB medium at 37°C until the OD600 reached 0.6. Protein expression was induced by adding 0.5 mM isopropylthio-β-D-galactoside (IPTG) for 4 h. The bacterial pellets were frozen overnight at -80°C to enhance cell lysis. After thawing, the pellets were lysed in MTTB buffer (50 mM Tris, 150 mM NaCl, 1% (w/v) Triton X-100, 0.2 mM phenylmethylsulfonyl fluoride (PMSF), aprotinin (5 mg/ml), leupeptin (10 mg/ml) and pepstatin A (1 mg/ml)). The protein lysates were incubated with GST beads for 4 h at 4°C. An aliquot of the E1B-55K-bound GST beads was boiled in 2x Laemmli loading buffer. To standardize protein concentration for the GST pulldown assay, an aliquot from each sample was analyzed via sodium dodecyl sulfate-polyacrylamide gel electrophoresis (SDS-PAGE) using a bovine serum albumin (BSA) standard curve to determine protein concentration. For the pulldown assay, cell lysates were prepared with RIPA buffer as described previously. 1 mg of protein lysate was incubated with amounts of GST-protein coupled beads, adjusted based on protein concentration, overnight followed by immunoblot analysis after boiling in 2x Laemmli loading buffer. Overnight incubation with GST-protein coupled beads also occurred at 4°C.

### 2.7 Indirect immunofluorescence

For indirect immunofluorescence, H1299 cells were seeded on glass coverslips at a density of 1.5 × 10^5^ cells per well. At various time points, the cells were fixed with 4% paraformaldehyde (PFA) for 20 minutes at 4°C and then permeabilized with PBS containing 0.5% Triton X-100 for 5 minutes at room temperature. After a 15-minute blocking step in Tris-buffered saline-BG (TBS-BG; BG consists of 5% (w/v) BSA and 5% (w/v) glycine), the coverslips were incubated with the indicated primary antibody diluted in PBS for 30 minutes (**Table 2**). The coverslips were then washed three times with TBS-BG, followed by a 20-minute incubation with Alexa 488 (Invitrogen) or Cy3 (Dianova)-conjugated secondary antibodies. Afterward, the coverslips were washed twice with TBS-BG and once with PBS. Finally, the coverslips were mounted using Glow medium (Energene), and digital images were captured using a confocal laser scanning microscope (Nikon). The images were cropped using Adobe Photoshop and assembled in Adobe Illustrator.

### 2.8 Statistical analyses

All statistical analyses were conducted using GraphPad Prism version 9. Details on the specific statistical tests used are provided in the corresponding figure legends. A *p* value of ≤0.05 was considered statistically significant.

## 3 Results

### 3.1 NF-κB promoter activation is counteracted during HAdV-C5 productive infection

**Figure 1:**
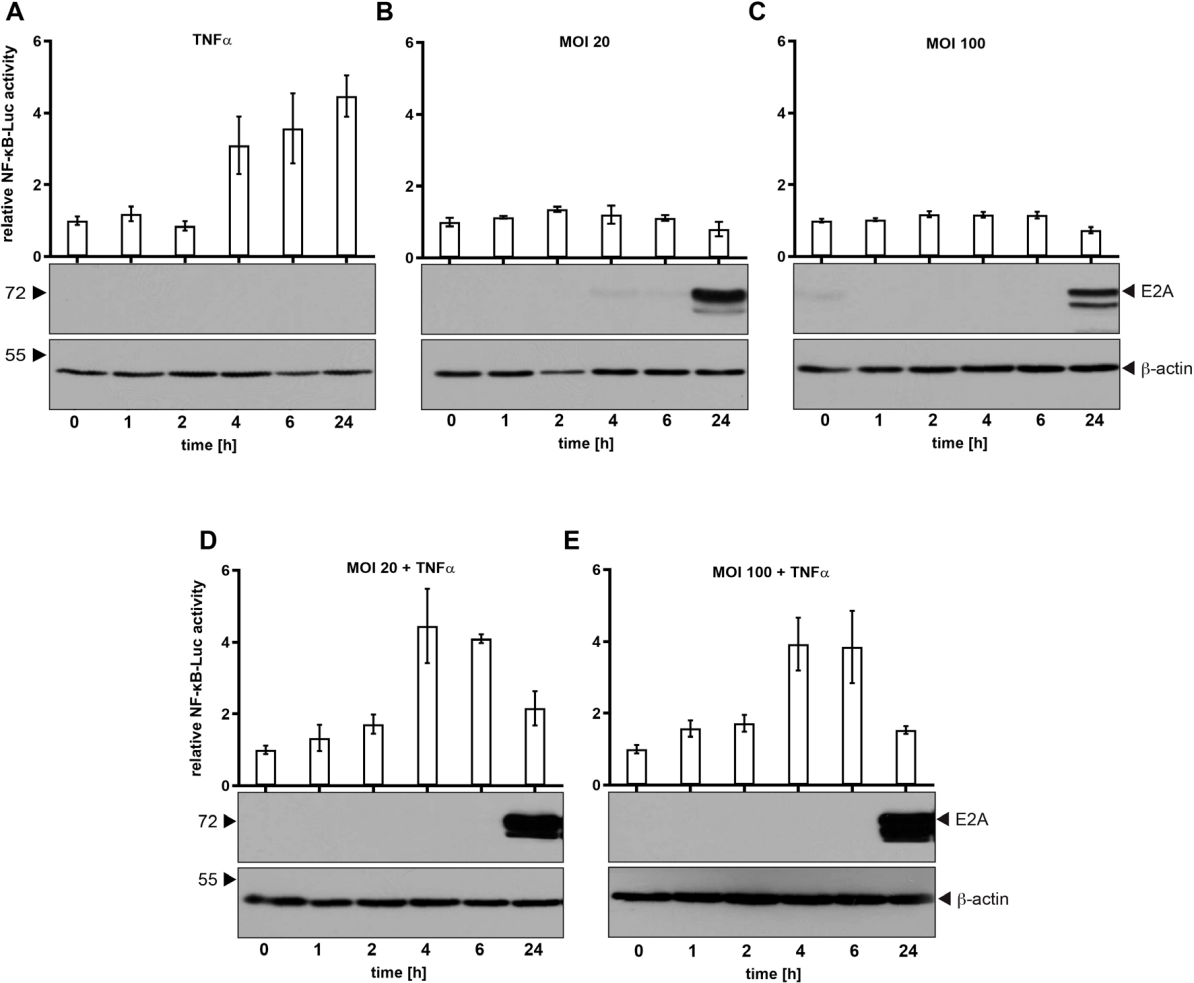
NF-κB promoter activity is not induced during HAdV-C5 productive infection. H1299 cells were transfected with 0.5 μg of the Renilla luciferase vector pRL-TK and 0.5 μg of the NF-κB reporter construct (5x-NF-κB-ELAM-promoter). **(A)** Cells were treated with 20 ng/ml TNFα and samples were collected and lysed at the indicated time points. **(B, C)** Cells were infected with HAdV-C5 wt (H5*pg*4100) at an MOI of either 20 or 100, as indicated. **(D, E)** Cells were treated with TNFα 2 h prior to infection and were subsequently infected for the indicated duration before lysis. Total cell extracts were prepared, and luciferase activity was measured. Firefly luciferase activity from the NF-κB-responsive reporter was normalized to Renilla luciferase activity to account for transfection efficiency. The mean and standard deviations from three technical replicates are presented. To assess adenoviral infection and ensure equal amounts of cell lysate were used, total cell lysates were resolved by SDS-PAGE and visualized via immunoblotting. E2A levels, used as an infection control, were detected with mAb B6-8 (α-E2A), while β-actin was detected with mAb AC-15 (α-β-actin) to serve as a loading control. Molecular weights (in kDa) are indicated on the left, with corresponding proteins labeled on the right.

It has been shown that proteins from the HAdV-C5 E3 region have immunomodulatory functions (29). Therefore, we used HAdV-C5, which does not encode the viral E3 region, to elucidate the role of HAdV-C5 proteins on NF-κB regulation during viral infection. We first investigated whether the NF-κB promoter is activated during a productive infection by HAdV-C5, comparing the activation levels to those triggered by TNFα, a representative of well-known activators of NF-κB signaling (30). The rationale behind this comparison is that TNFα, which is frequently secreted by macrophages or infected cells, binds to TNF receptors on nearby cells, thereby activating an immune response and preparing these cells to defend against potential infections. This approach allows us to assess NF-κB promoter activation in inflamed or primed cells (**Figure 1**). To control luciferase expression of the (5x) NF-κB-ELAM-promoter, the construct was transfected, and cells were treated for 24 h with 20 ng/ml TNFα 6 h post-transfection and harvested after indicated time points before cell lysis. H1299 cells treated with 20 ng/ml TNFα induce threefold luciferase expression after 4 h, raising up to 4.5 fold 24 h post-treatment (**Figure 1A**). However, H1299 cells infected with HAdV-C5 with an MOI of 20 or 100 did not show activation at any time point after infection. NF-κB activity is even slightly reduced at 24 h p.i. at MOI 20 and 100 compared to non-infected cells (**Figures 1B, C**). These results suggest that HAdV-C5 does not activate NF-κB in a manner similar to TNFα and it can be assumed that HAdV-C5 encodes for a protein that counteracts NF-κB activity. Thus, in the early phase of infection, the virus does not significantly disrupt the TNFα-induced activation of NF-κB. However, in the later phase of infection, the virus may interfere with TNFα activity.

In order to further investigate TNFα-mediated NF-κB activation, we combined infection with TNFα treatment. As shown, TNFα activates NF-κB 4 h p.i. The activity is not affected due to adenoviral infection at different virus concentrations (**Figures 1D, E**). Intriguingly, TNFα-mediated activation of NF-κB is decreased to the level of non-treated cells 24 h p.i. (**Figures 1D, E**). Therefore, we assume that HAdV-C5 encodes for a protein that counteracts this host defense mechanism.

### 3.2 NF-κB expression activates HAdV-C5 promoters

**Figure 2:**
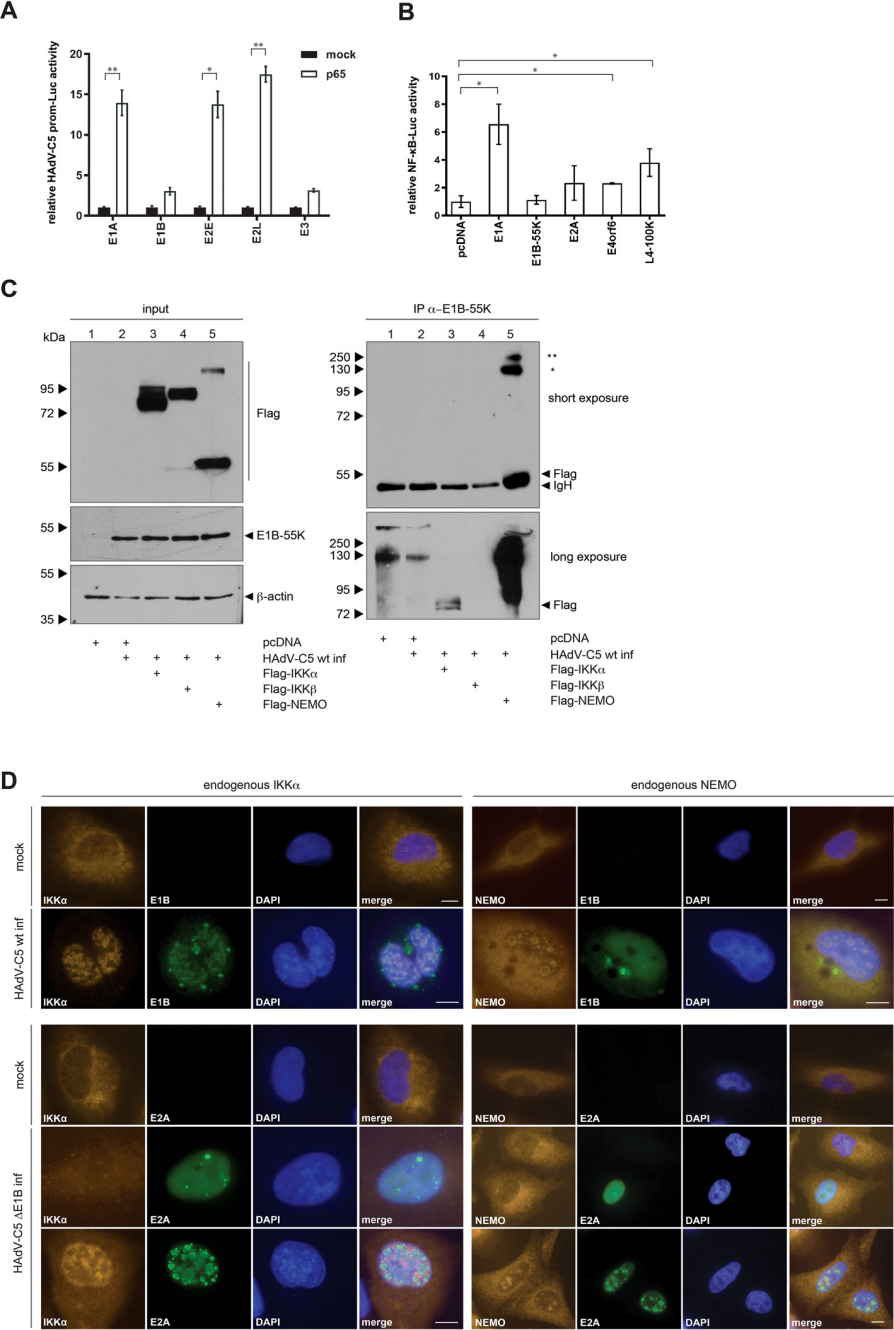
NF-κB overexpression activates HAdV-C5 promoters that modulate NF-κB activity. **(A)** H1299 cells were transfected with 1 μg of various HAdV-C5 promoter constructs, 0.5 μg of pRL-TK (Renilla-Luc), 0.5 μg of pGL3 basic, and 1 μg of p65. Whole-cell extracts were prepared, and luciferase activity was measured. Firefly luciferase activity for the HAdV-C5 promoter constructs was normalized to the corresponding Renilla luciferase activity, which served as an internal transfection control. The results are expressed as mean ± standard deviation from three independent experiments, indicating that HAdV-C5 proteins modulate NF-κB promoter activation. Asterisks indicate statistically significant differences (*p < 0.05, **p < 0.01; unpaired Student’s t-test). **(B)** H1299 cells were transfected with 0.5 μg of pRL-TK (Renilla luciferase), 0.5 μg of pGL3 basic 5x NF-κB-ELAM-prom as indicated, and 1 μg of a plasmid expressing viral proteins. Total protein extracts were prepared, and luciferase activity was determined. Firefly luciferase activity was normalized to that of the 5x-ELAM-prom construct expressed alone, along with its respective Renilla luciferase activity as a transfection efficiency control. The results are presented as mean ± standard deviation from three technical replicates. An asterisk indicates statistically significant differences (*p < 0.05; unpaired Student’s t-test). **(C)** Subconfluent H1299 cells were transfected with 5 μg of human Flag-tagged IKKα, IKKβ, and NEMO. The cells were infected 8 h post-transfection with H5*pg*4100 at an MOI of 20. Cells were harvested 24 h p.i., and whole-cell extracts were prepared. Co-precipitated proteins were visualized by immunoblotting. Input levels of total cell lysates were detected using mAb Flag-M2 (α-Flag), mAb 2A6 (α-E1B-55K), and mAb AC-15 (α-β-actin). Co-precipitated proteins were stained with mAb Flag-M2 (α-Flag). NEMO oligomerization is indicated by asterisks: (*) denotes NEMO dimers and (**) denotes NEMO trimers. Molecular weights are indicated on the left side of the panels, with corresponding protein labels on the right side. **(D)** HAdV-C5 induces nuclear relocalization of IKKα. A549 cells were infected with wt H5*pg*4100 (upper panel) or H5*pm*4149 (ΔE1B-55K; lower panel) for 24 h, fixed with 4% PFA, and double-labeled with mAb M-204 (α-IKKα) and mAb 2A6 (α-E1B-55K) or with E2A. Primary antibodies were detected using Cy3 (α-IKKα; orange) and Alexa488 (α-E1B-55K and α-E2A; green) conjugated secondary antibodies. 4′,6-diamidin-2-phenylindole (DAPI) was used for nuclear staining. Representative staining patterns for IKKα, α-E1B-55K, and α-E2A from at least 40 analyzed cells are shown, with overlays of individual images presented (magnification × 7600). Scale bars correspond to 10 µm.

Activation of NF-κB in response to viral pathogens is associated with the establishment of protective immunity, however the NF-κB pathway provides an attractive target to viruses as the rapid, immediate early (IE) event results in a strong transcriptional stimulation not only for cellular, but also for several early viral genes to enhance viral replication as they harbor NF-κB binding sites in their promoter sequences (3). NF-κB-binding sites have already been revealed within the promoter regions of HAdV-C5 E2 and E3 transcriptional units (31). This is associated with activation of the NF-κB pathway early after infection, which is beneficial for HAdV-C5 gene expression and replication. Here, we also investigated whether the early promoter regions of HAdV-C5 can be activated by the NF-κB transcription factor, with the ultimate goal of determining whether E1B-55K, as an adenoviral regulatory protein, interferes with this immune pathway – similar to the regulatory proteins of other viruses. H1299 cells were transfected with constructs encoding HAdV-C5 promoter sequences downstream of a reporter luciferase gene and co-transfected with a plasmid encoding p65, a subunit of NF-κB. As illustrated, p65 activates the E1A and the E2E promoter up to 14-fold, and the E2L promoter showed a 17-fold induction (**Figure 2A**). In contrast to the E1A, E2L and E3 promoter sequences, E2E has two NF-κB binding sites as shown by Machitani et al. (31). However, we observe strong E1A and E2L activation by p65 in contrast to E1B or E3 (**Figure 2A**). The lack of E3 promoter activation in our assay is notable, as it contradicts the current literature. However, these results suggest a highly regulated association between the cellular NF-κB pathway and the onset of adenoviral gene expression.

### 3.3 NF-κB promoter activity is highly regulated by transient expression of HAdV-C5 proteins

Viruses tightly regulate the immediate early step of immune activation in order to benefit from activation or inhibition of immune determinants. To elucidate the role of adenoviral proteins on NF-κB pathway regulation, H1299 cells were co-transfected with the NF-κB promoter construct and either early or late adenoviral gene encoding constructs (**Figure 2B**). The reporter gene expression construct used in this assay encodes a luciferase gene under the control of a (5x) NF-κB-ELAM-promoter. Firefly luciferase expression directly correlates with transcriptional activation by NF-κB. Renilla-luciferase was co-transfected to determine the transfection efficiency for normalization. This experiment shows that the early protein E1A increases the NF-κB promoter to 7-fold, while E2A, E4orf6, and the late protein L4-100K activate the NF-κB promoter 2- to 4-fold compared to the control. E1B-55K, however, showed no influence on the promoter activity. Thus, E1A appears to strongly activate the NFkB promoter, in contrast to E1B-55K (**Figure 2B**).

### 3.4 HAdV-C5 E1B-55K interferes with the IKK complex

Regulation of the NF-κB pathway by HAdVs could be maintained at several steps after infection at multiple NF-κB signaling molecules. Many viruses encode for viral proteins to target the NF-κB pathway. It has been reported that E1B-55K controls the expression of genes, which are involved in the NF-κB pathway and the regulation of interferon response genes (32). Building on these and our previous observations, we next examined the impact of HAdV-C5 infection on the formation of the IKK complex. H1299 cells were transfected with human Flag-tagged IKKα, IKKβ, NEMO plasmids and superinfected with HAdV-C5 wt at 8 h post-transfection. The western blot results show a higher migrating NEMO-specific band upon overexpression, indicating dimerization of the NEMO protein (**Figure 2C**, lane 5). Our results reveal interaction between E1B-55K with Flag-tagged IKKα and Flag-tagged NEMO (**Figure 2C**, lanes 3 and 5). These findings suggest a novel mechanism of NF-κB pathway modulation by the virus.

### 3.5 IKKα is relocalized into the nucleus and excluded from viral replication centers upon adenoviral infection

To investigate whether E1B-55K exerts an immune suppressive function by counteracting the NF-κB pathway through targeting the IKK proteins, we performed an immunofluorescence analysis. A549 cells were infected with HAdV-C5 and co-stained for E1B-55K and IKKα or NEMO. Consistent with previous publications, E1B-55K localizes within the cytoplasm and nucleus during infection (33, 34). Staining of E1B-55K reveals its nuclear localization with a few cytoplasmic nuclear membrane adjacent intensely stained bodies (perinuclear bodies) that is diffusely distributed (**Figure 2D**). Within the nucleus, E1B-55K shows mostly a granular diffuse distribution (**Figure 2D**). However, we also observed E1B-55K localization in globular structures, mostly associated globular ring-like structures (**Figure 2D**). Endogenous IKKα shows mainly cytoplasmic localization as it acts as member of the NF-κB signaling pathway within the cytoplasm, but a small proportion of IKKα is also detectable within the nucleus, likely to exert its NF-κB dependent as well as independent functions. However, IKKα shows a relocalization into the nucleus in infected cells (**Figure 2D**).

To elucidate the role of E1B-55K on IKKα localization in putative replication centers, H1299 cells were infected with an E1B-55K null mutant virus H5*pm*4149 and co-stained for the viral DNA binding protein (E2A) and IKKα. The early adenoviral protein E2A is a scaffold protein viral replication centers (35, 36) and serves as a control for infection (**Figure 2D**). Co-staining of IKKα and E2A reveals different localization pattern of IKKα early upon infection before constitution of viral replication centers in comparison to later time (**Figure 2D**). Early upon infection, which is indicated by a diffuse staining pattern of E2A, IKKα loses its preferred nuclear lamina localization and seems to be dispersed throughout the whole cell (**Figure 2D**). However, upon constitution of viral replication centers later upon infection, IKKα is mainly relocalized into the inter-viral replication center space of the nucleus (**Figure 2D**). Importantly, NEMO is partially relocalized to virus-induced nuclear globular compartments following adenovirus infection (**Figure 2D**). In the absence of infection, endogenous NEMO predominantly localizes in the cytoplasm. However, upon infection with HAdV-C5 wt, NEMO is partially relocalized to the nucleus, where it colocalizes with E1B-55K within the viral replication centers (as indicated by colocalization with DBP (36); **Figure 2D**). In summary, infection with an E1B-55K null mutant virus reveals E1B-55K independent relocalization of IKKα into the nucleus. This result does not only indicate that further viral factors could induce relocalization of IKKα, but also that there are further regulatory roles of E1B-55K on IKKα. Since E1B-55K null mutant virus infection also induces IKK relocalization, it suggests that other HAdV proteins may play a role in this effect, which warrants further investigation.

### 3.6 Characterization of the interaction between E1B-55K and IKK complex proteins

**Figure 3:**
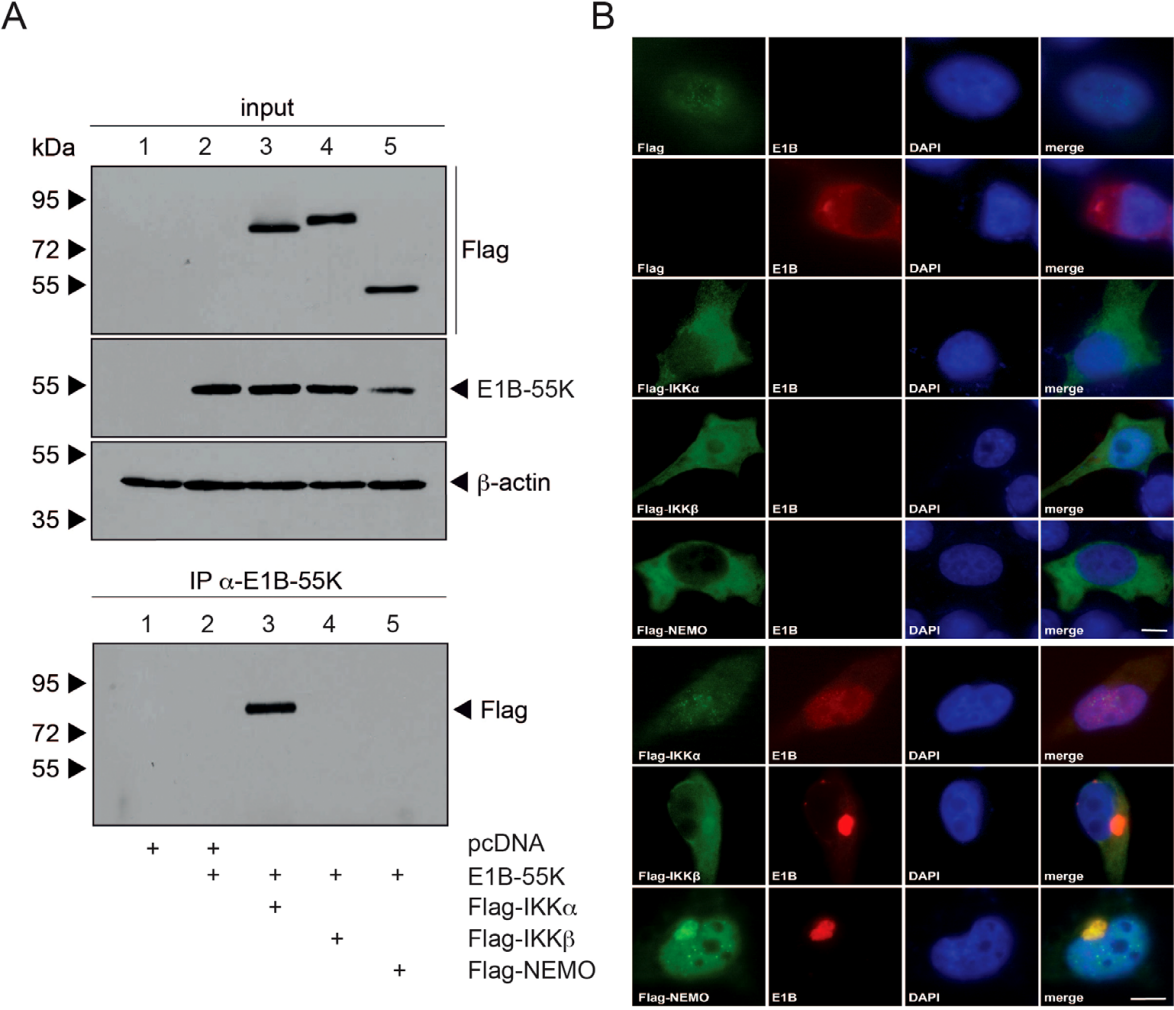
E1B-55K interaction with IKK complex components. **(A)** H1299 cells at subconfluent density were co-transfected with 5 µg each of E1B-55K, and Flag-tagged IKKα, IKKβ, and NEMO. At 48 h post-transfection, whole-cell extracts were prepared. Proteins were immunoprecipitated using the α-E1B-55K antibody 2A6, separated on an SDS-PAGE gel, and visualized by immunoblotting. For input controls, total cell lysates were analyzed using mAb Flag-M2 (α-Flag), mAb 2A6 (α-E1B-55K), mAb FL-419 (α-NEMO), and mAb AC-15 (α-β-actin). Immunoblotting of co-precipitated proteins was conducted with mAb Flag-M2 (α-Flag). Molecular weights are marked on the left, with protein labels on the right. **(B)** H1299 cells were transfected with Flag-tagged IKKα, IKKβ, or NEMO, along with E1B-55K as indicated. At 48 h post-transfection, cells were fixed with 4% PFA and double-labeled using mAb 2A6 (α-E1B-55K) and mAb M2 (α-Flag). Primary antibodies were detected using Alexa 488-conjugated (α-Flag) and Texas Red-conjugated (α-E1B-55K) secondary antibodies. Nuclear staining was performed with DAPI. Representative staining patterns for α-E1B-55K and α-Flag from at least 29 cells are shown, with merged images provided (magnification × 7600). Scale bars correspond to 10 µm.

Next, we aimed to further analyze the interaction between E1B-55K and IKK proteins through transfection experiments to determine whether this interaction occurs independently of other viral proteins. We repeated immunoprecipitation experiments with E1B-55K, co-transfecting it with each of the Flag-tagged IKK proteins in H1299 cells (**Figure 3A**). Input samples for direct immunoprecipitation of E1B-55K showed sufficient and comparable levels of both transfected and endogenous proteins. Our findings indicate that E1B-55K may interact with IKKα but not with NEMO, unlike in a full viral infection (**Figures 2C**, **3A**). This suggests that additional viral proteins may be required for interaction with NEMO.

To investigate these interactions in more detail, we conducted immunofluorescence analysis. For this, H1299 cells were co-transfected similarly to the immunoprecipitation experiments, using Flag-tagged IKKα, IKKβ, NEMO, and E1B-55K. Cells were then fixed with PFA 48 hours post-transfection and visualized through double-labeled immunofluorescence microscopy (**Figure 3B**). In cells transfected only with Flag-tagged E1B-55K or IKKα, both proteins predominantly showed cytoplasmic localization. However, upon co-transfection of both proteins, E1B-55K was almost entirely relocalized diffusely within the nucleus, while Flag-tagged IKKα lost its cytoplasmic localization and became diffusely distributed throughout the entire cell (**Figure 3B**). Interestingly, upon co-transfection with E1B-55K, both IKKβ and NEMO showed complete relocalization into the nucleus and perinuclear bodies where they colocalized with E1B-55K (**Figure 3B**).

### 3.7 Mapping the interaction domains of E1B-55K with IKKα

**Figure 4:**
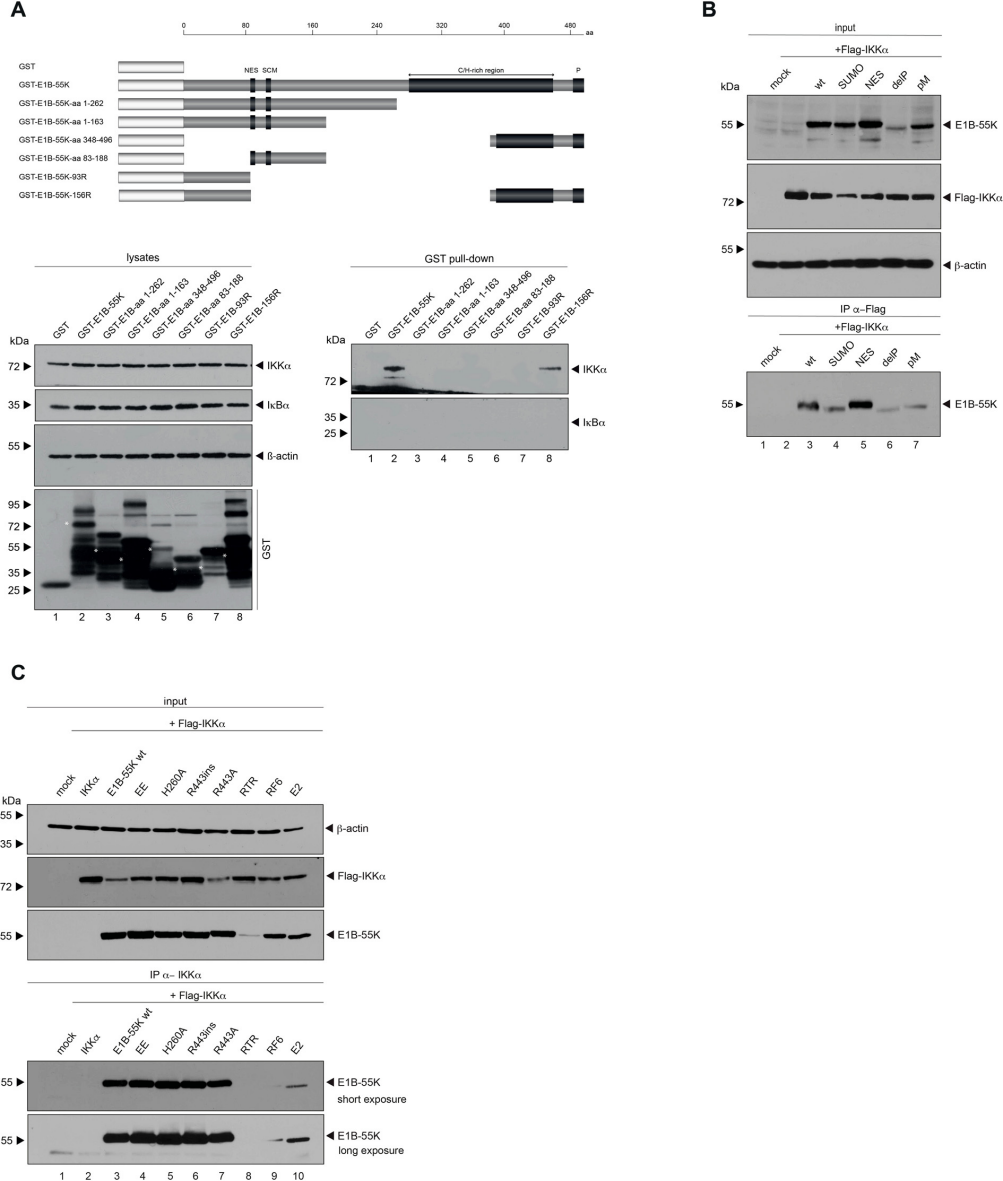
In depth characterization of binding between E1B-55K and IKKα. **(A)** GST-tagged truncation mutants of E1B-55K, expressed in *E. coli*, were conjugated to glutathione sepharose beads. These conjugates were incubated with 750 μg of protein lysates, separated by SDS-PAGE, and immunoblotted using Ab M-204 (α-IKKα) for visualization. As a control, a duplicate experiment was stained with Ab C-21 (α-IκBα). Input levels of total-cell lysates were detected with Ab M-204 (α-IKKα), Ab C-21 (α-IκBα), and mAb AC-15 (α-β-actin). White asterisks indicate E1B-55Ks. **(B)** Subconfluent H1299 cells were co-transfected with 5 μg of either wt E1B-55K or specified E1B-55K mutants, along with 5 μg of human Flag-tagged IKKα. Cells were harvested at 48 h post-transfection for whole-cell extract preparation. Immunoprecipitation of Flag-tagged IKKα was carried out using mAb M2 (α-Flag), followed by SDS-PAGE separation and immunoblotting. Input levels of whole-cell lysates were detected using mAb Flag-M2 (α-Flag), 7C11 (α-E1B-55K), and mAb AC-15 (α-β-actin). Co-precipitated proteins were stained 7C11 (α-E1B-55K). **(C)** Subconfluent H1299 cells were co-transfected with 5 μg of either wt E1B-55K or specific E1B-55K mutants, along with 5 μg of human Flag-tagged IKKα. Cells were harvested 48 h post-transfection, and total-cell extracts were prepared. Flag-tagged IKKα was immunoprecipitated with mAb M2 (α-Flag), followed by SDS-PAGE separation and immunoblotting. Input levels of total-cell lysates were detected using mAb Flag-M2 (α-Flag), mAb 2A6 (α-E1B-55K), and mAb AC-15 (α-β-actin). Co-precipitated proteins were visualized with mAb 2A6 (α-E1B-55K). Molecular weights are indicated on the left, with corresponding proteins labeled on the right side of the panels. aa, amino acid; NES, nuclear export signal; SCM, SUMO conjugation motif; C/H-rich region, cysteine/histidine-rich region; P, phosphorylation sites.

To pinpoint which regions of E1B-55K are responsible for interacting with IKKα, we conducted GST pulldown assays. Therefore, bacterial expressed GST-tagged E1B-55K wt, truncation mutants as well as isoforms of E1B-55K (**Figure 4A**) were purified and conjugated to glutathione sepharose beads. The E1B-55K mutants we used have already been extensively studied, allowing us to explore potential functions or mechanisms. Cell lysates were incubated with purified GST-tagged protein conjugated beads and an interaction was analyzed by SDS-PAGE and Western blotting. GST pulldown experiments confirm the interaction between IKKα and E1B-55K. Additionally, IKKα interacts with the isoform 156R of E1B-55K (**Figure 4A**), which shares the same N- and C-terminus of E1B-55K but misses the central part. This isoform lacks both the NES and the SUMO conjugation motif (SCM), resulting in its localization to the cytoplasm. However, like wt E1B-55K, 156R is phosphorylated at its C-terminus. These findings suggest that the central region of E1B-55K is not essential for its interaction with IKKα, but that protein folding or phosphorylation of E1B-55K may play a crucial role in enabling this interaction.

In the past, numerous efforts were made to characterize E1B-55K by generating various single-amino-acid substitution mutants, as well as multiple amino-acid substitutions, to disrupt specific motifs or PTMs such as SUMOylation or phosphorylation, resulting in the regulation of protein-protein interactions (19, 20, 26). Consequently, we investigated whether these PTMs of E1B-55K are essential for its interaction with IKKα by co-immunoprecipitation experiments. Our findings strongly indicated that all E1B-55K PTM mutants tested still bind IKKα (**Figure 4B**). However, we also we found that IKKα interaction was impaired in specific E1B-55K mutants (RF6 and E2, that have previously been reported to show defects in binding to the PML-NB factors Mre11 (E1B-55K-RF6) and Daxx (E1B-55K-E2); (37); **Figure 4C**), indicating that point mutations or structural differences in these mutants, but not PTMs disrupt the interaction and suggesting a degree of flexibility in the interaction.

### 3.8 IKKα supports adenovirus progeny production and protein expression

**Figure 5:**
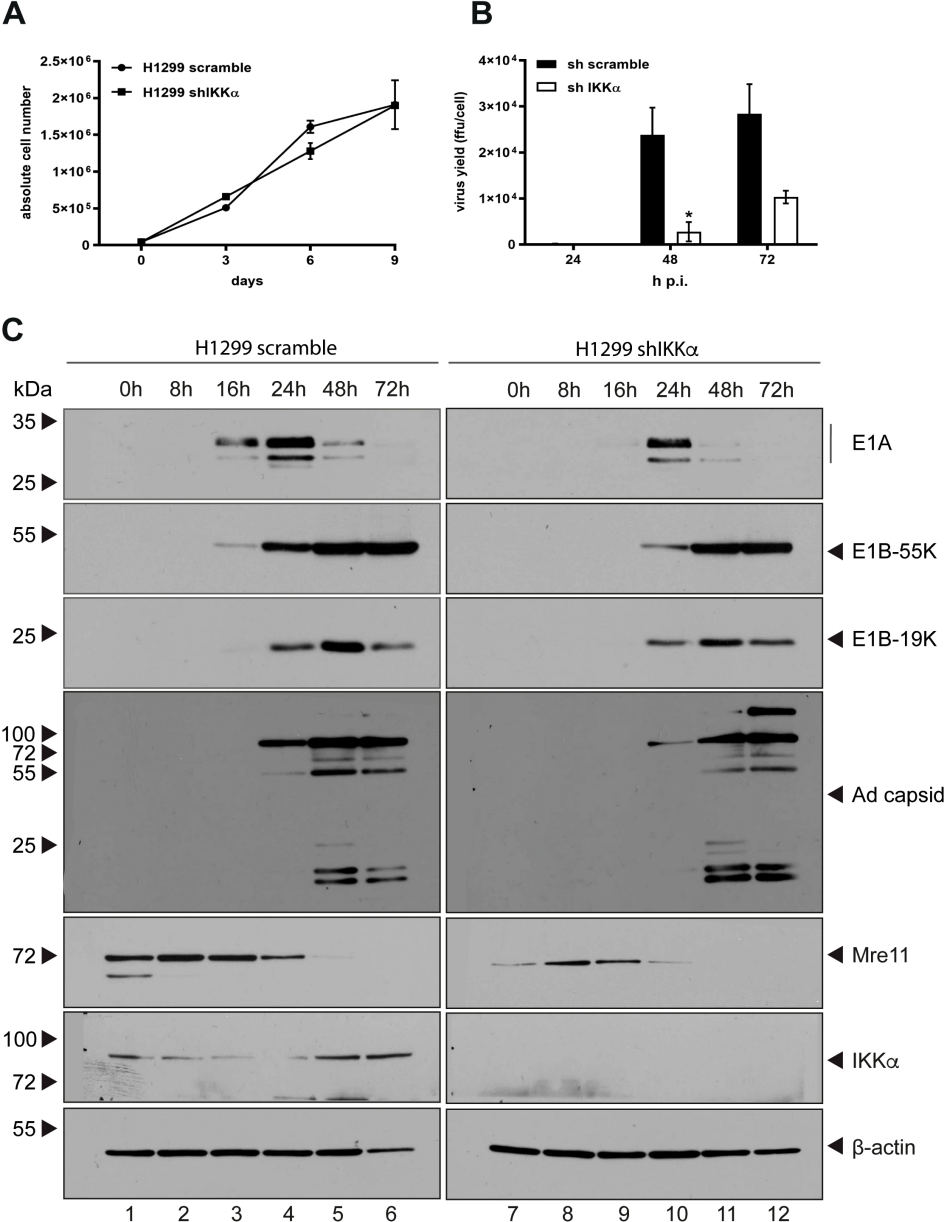
Depletion of IKKα enhances production of HAdV-C5 progeny. **(A)** A total of 1 × 10_4_ cells were cultured, and growth was monitored by measuring absolute cell counts at the indicated time points. Data represent the mean of from two independent experiments. Error bars indicate standard deviations. **(B)** H1299 parental and shIKKα knockdown cells were infected with wt H5*pg*4100 at an MOI of 20. Viral particles were collected at 24, 48, and 72 h p.i., and virus yields were quantified via E2A immunofluorescence staining. Results are averages from three independent experiments, with error bars representing the standard deviations. An asterisk indicates statistically significant differences (*p < 0.05; unpaired Student’s t-test). **(C)** H1299 parental and shIKKα cells were infected with wt H5pg4100 at an MOI of 20. Total-cell extracts were collected, and proteins were separated by SDS-PAGE and analyzed by immunoblotting. The following antibodies were used: α-Mre11 (rabbit), α-IKKα (M-204), α-β-actin (AC-15), α-E1A (mouse, M-58), α-E2A (B6-8), α-E1B-55K (2A6), and HAdV-C5 capsid proteins (rabbit antiserum L133). Molecular weights are indicated on the left, and corresponding proteins are labeled on the right side of each panel.

Viruses modulate their hosts in order to achieve a microenvironment, which promote productive viral infection. Therefore, cells have to counteract anti-viral defense measurements by either being targeted to protein degradation, subcellular localization changes or they are inactivated by modulation of their PTMs. To analyze the effect of IKKα on HAdV-C5 progeny production, lentiviral particles harboring shRNA (shIKKα) was transduced to deplete endogenous IKKα from H1299 cells prior to H5*pg*4100 virus infection. IKKα was efficiently depleted as shown with western blot analysis (see below). Growth curve analysis indicates, that the knockdown of IKKα did not affect the growth rate of the cells (**Figure 5A**). In comparison to the parental cell lines, which were transduced with lentiviral particles harboring scrambled shRNA, knockdown of IKKα decreased the production of infectious virus particles at 8.62-fold at 48 h p.i. and 2.76-fold at 72 h p.i. (**Figure 5B**). Our data indicate that IKKα acts as a negative regulator of HAdV-C5 progeny production.

Next, the role of IKKα on transcriptional regulation of adenoviral genes was investigated. In order to analyze the role of IKKα on viral early and late protein expression in H1299 cells, western blot analysis was made to monitor viral protein expression levels at different time points after infection. Consistent with affected HAdV-C5 progeny production, expression of particular HAdV-C5 early proteins E1A and E1B-55K are decreased in IKKα depleted cells compared to the parental cells and protein steady state levels of E1B-19K and adenoviral capsids are similarly reduced at 24 hpi, comparable to the reduction observed in E1A and E1B-55K. Moreover, the Mre11 steady state levels are higher in the parental cells compared to the shIKKα knockdown cells throughout the course of infection (**Figure 5C**). Taken together, these data indicate that IKKα is a positive regulator of HAdV-C5 replication during infection. Additionally, the data suggest that IKKα likely influences the DNA damage response through Mre11.

## 4 Discussion

The findings presented in this study reveal a novel mechanism by which HAdV-C5 modulates the host immune response, particularly through targeting the IKK complex during infection. Our results indicate that HAdV-C5 infection inhibits TNFα-mediated NF-κB activation by relocating IKKα to the nucleus (and, thereby, presumably disrupting IKK complex formation), where it supports viral replication. In addition to the known finding that HAdVs utilize NF-κB during the early phase of infection for efficient replication, we demonstrate that TNFα-induced immune responses via the NF-κB pathway are repressed by E1B-55K during the late phase of replication. Furthermore, the virus appears to exploit NF-κB pathway-independent functions of IKK complex proteins for replication, as knockdown of IKKα results in reduced viral replication. This biphasic regulation is comparable to findings from cytomegalovirus studies, which showed that, after an initial transient phase of activation, a cytomegalovirus protein blocks NF-κB-activating pathways by inducing IKK complex degradation (38, 39). It is well-established that many viruses encode proteins to manipulate the NF-κB pathway to their advantage. For example, hepatitis C virus (HCV) produces proteases NS3-NS4, which degrade TRIF, a key protein in TLR9-mediated NF-κB activation (40). Besides, HCV core proteins and NS5B inhibit the IKK complex by directly interacting with IKK components (41, 42). As shown here for HAdVs, certain other viruses also modulate NF-κB to prevent apoptosis, thereby prolonging cell survival and facilitating viral replication. For instance, human herpesvirus 8 (HHV-8) encodes vFLIP, an activator of NF-κB, which prevents apoptosis in infected cells (43). Conversely, the coxsackievirus protease 3Cpro inhibits NF-κB to promote apoptosis, effectively prolonging infection by suppressing host immune defenses (44). Based on the results of our luciferase assays (**Figure 2B**) and the co-localization of NEMO with DBP (**Figure 2D**), future studies should investigate how these proteins interact with the IKK complex and whether their regulation is influenced by different viral proteins. Since E1B-55K is a viral oncoprotein, it might be worthwhile to investigate whether E1B-55K could potentially transform cells by targeting the IKK complex in future studies. Such viral strategies illustrate how viruses selectively inhibit or activate NF-κB through the IKK complex to benefit their replication or evade host immune responses.

NF-κB activation is a critical early immune response to infection and plays a dual role for viruses. On one hand, NF-κB triggers protective immunity; on the other, it can promote viral replication. By initiating a strong transcriptional response, NF-κB not only induces host immune genes but can also enhance the expression of viral genes that contain NF-κB binding sites, as seen in retroviruses (e.g., HIV), HAdVs, papovaviruses, and herpesviruses (3, 45–52).

HAdV infection triggers an early immune response as viral fibers bind to the CAR receptor, initiating ERK1/2, JNK, and MAPK signaling, which subsequently leads to NF-κB activation and chemokine production (53–55). This pathway also stimulates IL-10 production, promoting an anti-inflammatory Th2-type response, inducing cytokines such as IL-4 and IL-13 (56). However, in wt HAdV infections, IFN production is suppressed by the immediate-early protein E1A, which interferes with IFN-responsive gene expression and inhibits JAK/STAT signaling (53, 57–64). The IFN response is also blocked at a later stage of infection. This is mediated by the virus-associated (VA) RNAs. Upon HAdV infection, VA-RNA can regulate the IFN response by preventing PKR activation, affecting this way the inflammatory response and apoptosis (65, 66). Apart from regulating IFN, HAdV infection induces TNF in infected tissues (67). However, the E1A, E1B, and E3 proteins modulate TNF signaling, as the absence of these proteins sensitizes cells to TNF-dependent lysis (68–71). Shao et al. showed that E1A indirectly inhibits NF-κB activation by modifying IKK-mediated IκB phosphorylation, thereby preventing NF-κB nuclear translocation and TNF-induced apoptosis (72). Notably, the influence of E1A on NF-κB is complex and varies with cell type, infection phase, additional viral factors, and experimental setup (71–73). Additionally, HAdV encodes four proteins – E1B/19K and E3 proteins (14.7K, 10.4K, and 14.5K) – that counteract TNF-mediated cytolysis, further supporting immune evasion and prolonged infection (74, 75). Our results identify another adenoviral protein involved in modulation and suggest that its regulation is similar to that of p53, with multiple adenoviral proteins contributing to the interference with this critical apoptosis pathway – potentially indicating that the virus relies on diverse mechanisms to deregulate this pathway.

In addition, human tumor viruses such as HTLV-1 and EBV also utilize the NF-κB pathway to support their transforming abilities. The HTLV-1 Tax protein associates with NEMO to form stable complexes that lead to chronic NF-κB activation, continuous IκB turnover, and persistent NF-κB expression (76). EBV LMP1, in contrast, activates NF-κB, initiating a cascade that includes NIK and IKK activation (77). These interactions illustrate how oncogenic viruses – including HAdVs (?) – exploit NF-κB to maintain (persistent) infections and promote cell transformation.

These results suggest that, in the early phase of infection, the virus does not significantly disrupt the TNFα-induced activation of NF-κB. However, in the later phase of infection, the virus may interfere with TNFα activity. In addition, our findings reveal a new mechanism through which HAdVs suppress immune signaling by disrupting activation of the NF-κB pathway, specifically by blocking IKK formation. Additionally, we observed that E1B-55K, a multifunctional oncoprotein of HAdVs (19, 78), interacts with components of this pathway in the cell nucleus. The observed interactions between E1B and IKKα, along with the altered localization of IKKα, IKKβ, and NEMO during infection, suggest potential implications for the classical NF-κB pathway and support our proposed model in which E1B-55K suppresses NF-κB activation. Moreover, the interaction of E1B-55K with IKKα promotes viral replication while likely helping the virus evade detection by the immune system. Future studies should explore whether the knockdown of other IKK complex components also inhibits virus production. Additionally, it can be hypothesized that E1B-55K expression may indirectly cause the relocalization of IKKβ and NEMO by deregulating specific cellular proteins after its expression.

In conclusion, our study highlights the sophisticated strategy HAdV-C5 employs to manipulate the host NF-κB pathway by targeting the IKK complex, facilitating viral replication while evading immune defenses. Understanding these interactions provides valuable insights into adenoviral pathogenesis and can inform the development of targeted antiviral therapies.

## Data Availability

The original contributions presented in the study are included in the article. Further inquiries can be directed to the corresponding authors.
